# A Novel Method for Improving Weld Seam Properties through Inline Coupling of Welding and Forming

**DOI:** 10.3390/ma13020271

**Published:** 2020-01-07

**Authors:** Sebastian Härtel, Tom-Eric Adams, Kevin Hoefer, Birgit Awiszus, Peter Mayr

**Affiliations:** 1 Department of Mechanical Engineering, Chemnitz University of Technology, 09111 Chemnitz, Germany; kevin.hoefer@mb.tu-chemnitz.de (K.H.); birgit.awiszus@mb.tu-chemnitz.de (B.A.); 2 Department of Mechanical Engineering, Technical University of Munich, 80333 München, Germany; tom.adams@tum.de (T.-E.A.); peter.mayr@tum.de (P.M.)

**Keywords:** WeldForming, post weld treatment, recrystallization, thermomechanical simulation, numerical simulation, FEM

## Abstract

High heat input during welding leads to transformations of the microstructure in the area subjected to welding, mostly resulting in an inhomogeneous microstructure and an overall deterioration of the mechanical properties. To restore these properties, post treatment processes which are typically separated from the welding process are state of the art. The present work focusses on recrystallization phenomena and shows the new methodology, WeldForming, which intends to eliminate subsequent treatment processes. The new inline process combination harnesses the synergies of a welding and a rolling process to ultimately prevent the typical zone formation of the heat affected zone. This is done by stimulating recrystallization and recovery processes. The development of the process is based on numerical simulations. A new aspect is the first time coupling of both processes in a single simulation model. The required material models were generated with the aid of thermophysical simulations. A novel approach is shown for creating a material model for the filler metal G4Si1 with the typical directed solidification microstructure. On the basis of the gained knowledge out of thermomechanical and numerical simulation, a process window was identified and a test setup was developed which gave the functional prove of the WeldForming process.

## 1. Introduction

### 1.1. State of the Art

Up until today welding is a widely used process for joining sheet metal and various semi-finished products. The standard DIN 8593-6 defines welding as a process in which two or more parts are joined by a suitable application of heat and/ or pressure. The selection of a suitable welding process is mainly dependent on the present geometry and the materials to be joined.

Energy input is a vital part of every welding process. Particularly in fusion welding this energy input in the form of heat may lead to undesired phase transformations and microstructural changes which most likely lead to a deterioration of the mechanical properties.

**Figure 1:**
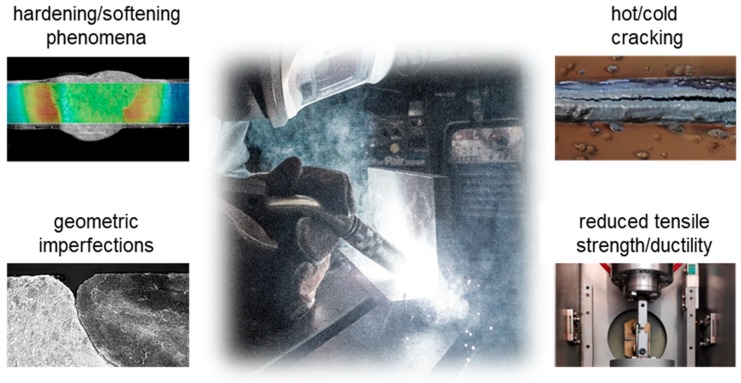
Overview of Defects and irregularities in the welding area.

This is especially relevant for steel welds as stated by Ashby and Easterling [1] and Ion et al. [2]. Figure 1 shows an overview of typical defects and irregularities, which result directly or indirectly out of the energy input and cooling behavior. The characteristic temperature profile of the welding process with its distinct temperature gradients and cooling rates, can cause a variety of unwanted phenomena, like segregations or different microstructural features after solidification.

A critical part of the joining area is the zone next to the fusion line. This zone is called the heat-affected-zone (HAZ). The HAZ thereby describes the zones of the joining area where the base material is altered by the welding thermal cycle but not melted. Kojima et al. [3] describe in their work that, if for example gas metal arc welding (GMAW), a high heat input process, is used for welding steels, the microstructure close to the fusion line becomes notably coarse and the tensile strength of this zone is highly degraded.

As commonly stated, the HAZ consist out of a grain growth, a recrystallized, a partially transformed and a tempered zone, each zone with its own characteristic mechanical properties. These varying properties can lead to a weakening of the whole joint, e.g. inferior strength relative to the unaffected base material, as mentioned in research done by the Sodel LTD [4]. In the work of Mohandas et al. [5] various experiments on high-strength low-alloy steels were conducted and the effect of the microstructural transformations in the HAZ on the overall properties of the assembly were analyzed.

**Figure 2:**
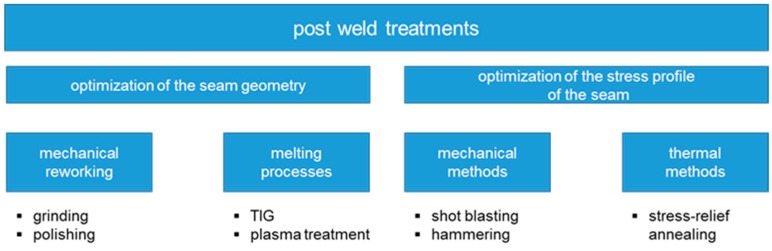
Classification of post weld treatments.

To reduce the negative effects of the welding process a large variety of after treatment processes are available. Kirkhope et al. [6] divided the weld fatigue improvement methods into two main groups. The first group aims at geometry modification methods that remove weld toe defects and / or reduce the stress concentration. Processes of the second group serve to optimize the stress profile of the weld by reheating or by introducing a compressive stress field in the area where cracks are likely to initiate. Examples for different after treatment processes are shown in Figure 2.

In a number of investigations done by Haibach [7], Booth [8], Bignonnet [9] and Haagensen [10] an improvement of the fatigue strength after mechanical reworking processes like grinding or polishing could be proven. An alternative to reworking processes are melting processes like TIG (Tungsten Inert-Gaswelding) and plasma treatments. By a subsequent melting, undercuts and near surface micro cracks in the weld seam transition can be reduced by rounding out these areas. The influence of the TIG melting process on the fatigue strength was quantified by Kado et al. [11] and Ikeda et al. [12]. An improvement in fatigue strength of 10 % was achieved for butt welds made of low-alloy steels and 140% for fillet welds made of high-strength steels.

In addition to the above-mentioned processes, which optimize the seam geometry, the second main group uses mechanical and thermal treatment processes (shot peening and hammering, stress-relief annealing) to optimize the stress profile in the weld. Mechanical processes like hammering are performed by mechanical hammering with a bolt at a frequency of approx. 200 Hz and an amplitude of 40 μm. By near-surface plasticizing of the weld seam, residual compressive stresses are induced, which lead to improved fatigue properties by suppressing the growth of cracks. Investigations by Bremen [13] show that residual compressive stresses can be applied to a depth of 4.0 mm during hammering and up to 1.5 mm during needling. The residual compressive stress introduced by hammering in the course of these investigations amounted to approx. 65% of the yield strength and by needling approx. 90% of the yield strength. The thermal method of stress relief annealing is also an adequate measure to improve the fatigue behavior of the weld by reducing the residual stresses or locally increased hardness values, which result out of the welding process, as stated by Müsgen [14].

### 1.2. Process Principle of WeldForming

**Figure 3:**
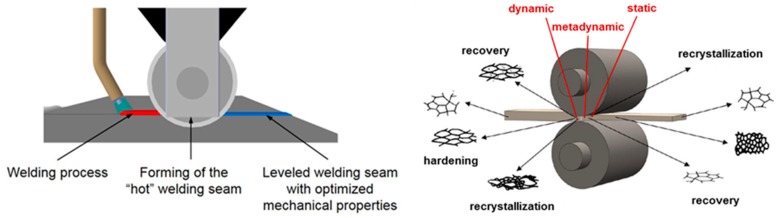
Schematic of the weld-forming process (**left**); softening mechanisms during and after hot rolling (**right**), according to Ullmann [15].

The disadvantage of current post-treatment methods is that they are separated from the actual welding process, thus requiring additional process steps. The new methodology of WeldForming intends to solve this problem and eliminate the decoupled treatment processes. The goal of WeldForming is a purposeful altering of the microstructure, restoring the mechanical properties of the joint which were degraded by the welding process. Recrystallization and recovery processes provide for a change of the typical anisotropic texture of the welding zone to a homogenous base material-like microstructure. By coupling a welding and a rolling process it is possible to utilize the thermal energy of the welding process to initiate above mentioned processes. The rolling process induces a thermal microstructure optimization. Additionally, a leveling of the weld seam to sheet thickness is achieved. This in turn reduces critical notches and leads to an increase of the dynamic strength of the assembly. A schematic sketch of the WeldForming process and the different softening mechanisms are shown in Figure 3.

## 2. Materials and Methods

For the welding process the steel grade S235JR (1.0037) was used as base material and for the necessary filler material the higher alloyed steel G4Si1 (1.5130) was used. In Table 1 shows the chemical composition for the used materials.

A stable process window for the welding process had to be determined prior to the process development of WeldForming. One of the main requirements of the welding process was to achieve a high face reinforcement to maximize the plastic strain in the subsequent forming process.

Two S235 steel plates with an initial thickness of 4.0 mm were welded together in a butt joint configuration. For the realization of the welding tests a FroniusTransPuls Synergic 5000 CMT R welding power source (FRONIUS INTERNATIONAL GMBH, Pettenbach, Austria) with a connected VR 700 CMT (FRONIUS INTERNATIONAL GMBH, Pettenbach, Austria) wire feeder was used. Furthermore, the guidance of the welding torch was managed by a six-axis articulated arm robot (Comau S.p.A., Grugliasco, Italy) in order to ensure the highest possible reproducibility.

In Härtel and Adams [16], the effect of the welding speed on the seam geometry has already been investigated extensively. It was found that a welding speed of 0.9 m/min is suitable for producing a sufficient weld reinforcement and for inducing sufficient thermal energy. A high face and root reinforcement results in a high plastic strain when forming the seam to sheet thickness and thus promotes recrystallization. Furthermore, a high energy input by the welding process leads to a higher rolling temperature which favors the initiation of recrystallization effects.

**Figure 4:**
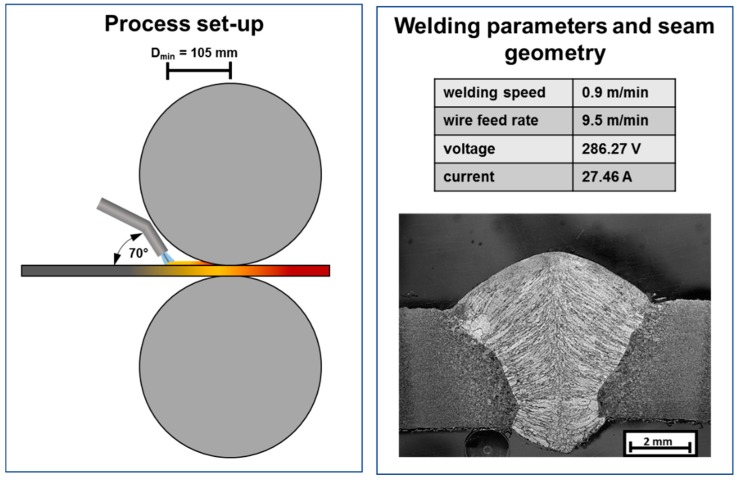
Process set-up for WeldForming **(left**) and welding parameters with corresponding cross section of the weld seam without additional rolling (**right**).

In addition to the welding process, the distance between the welding torch and the roll center is also decisive. In order to keep the distance between the welding torch and the roller center as small as possible, the welding torch is set at an angle of 70°. This makes it possible to investigate the distance between the welding torch and the center of the roll to a larger extent. Welding torch configuration and process parameters are shown in Figure 4.

## 3. Process Window and Model Parameter Estimation

### 3.1. Experimental Setup

**Figure 5:**
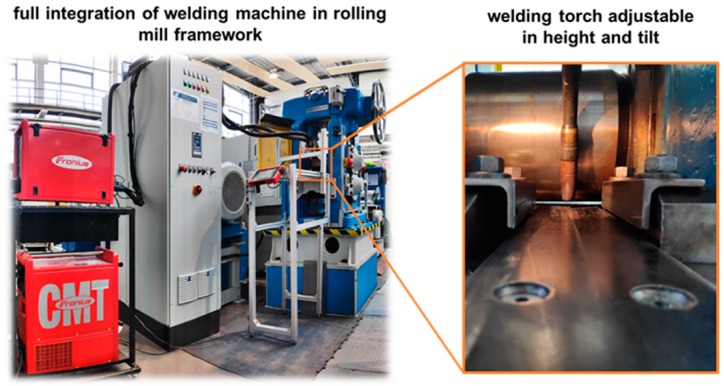
Experimental setup for the WeldForming process.

In order to implement the welding process experimentally, a corresponding test setup was built on a laboratory scale (see Figure 5). For this purpose, an existing duo reversing cold rolling mill was extended by a water-cooled welding torch from Fronius. The constructive integration of the welding torch into the rolling stand was carried out by means of aluminum hollow profiles. The welding torch is integrated in such a way that the distance between the welding torch and the roll center can be adjusted between 105 mm and 300 mm at a torch angle of 70°. Already in Härtel and Adams [16] suitable welding parameters were investigated at a torch angle of 70°. These parameters were also used for the investigations in the present work. However, the focus of this work lies on the influence of the distance, welding torch to roll center, on the recrystallization behavior in the weld metal and coarse grained HAZ as well as their resulting mechanical properties. The basic idea is that a short distance from torch to roll leads to a higher rolling temperature because the seam has less time to cool down. Subsequently, the interactions between the torch to roller distance and the resulting microstructure and thus mechanical properties are investigated experimentally and numerically.

### 3.2. Process Limits

**Figure 6:**
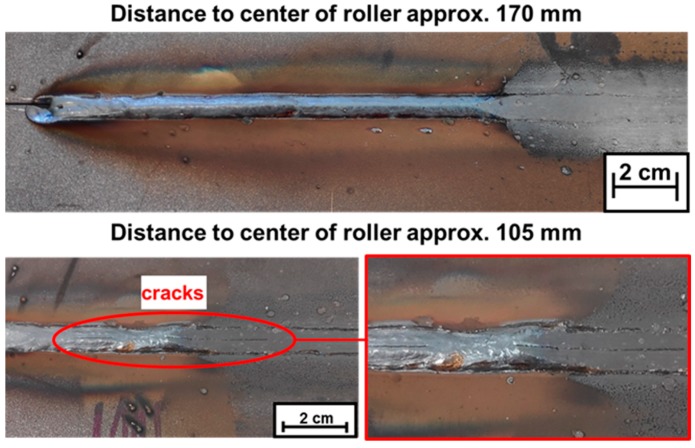
Samples after WeldForming with different distances between torch and roller.

In a first step, the WeldForming process was performed by varying the distance between the welding torch and the roll center. Subsequently, an optical inspection of the components for possible connection errors was carried out. Figure 6 shows the welded and rolled components for a torch to roller distance of 170 mm and 105 mm. The distances of 170 mm and 130 mm lead to joints that are free from defects. At a distance of 105 mm, on the other hand, a longitudinal crack occurs in the weld seam. The reason for the crack formation lies in the higher weld metal temperature at a distance of 105 mm. This was measured with a pyrometer 50 mm before the center of the roller and lies at approx. 1250 °C.

At this higher temperature the solidifying weld seam does not have the necessary strength to withstand the stresses that occur. Therefore, the residual stresses and the high cooling rate caused by the current forming process probably lead to cracks along the center rib of the seam prior to forming, as shown in Figure 6. Since the strength of the seam is significantly reduced due to the high temperatures, the cracks also occur in the non-formed areas.

### 3.3. Microstructural Investigations

**Figure 7:**
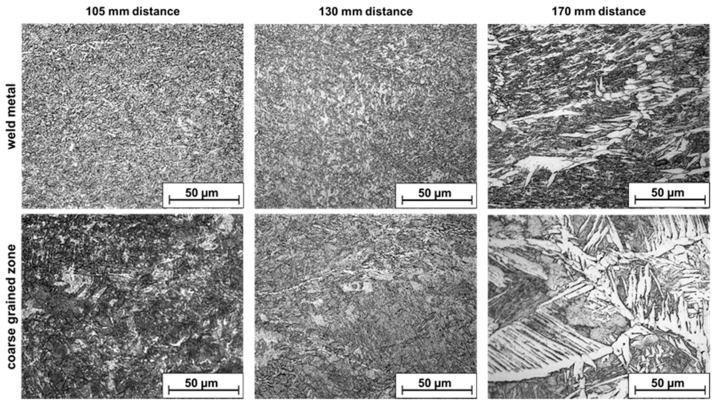
Microstructure development of the weld metal and the coarse-grained HAZ by varying the torch to roller distance.

Light microscopy and SEM (scanning electron microscope) imaging was used to analyze the transformation of the microstructure in the seam area. The investigated area is identical to that in the later simulation and located in the center of the sample. Figure 7 shows microstructural images the weld metal and the coarse-grained HAZ of a weldformed sample. The typical cast-like structure of the weld metal, which is still present in the sample produced with a torch to roller distance of 170 mm (Figure 7, top right) can be transformed into a fine grained structure by means of recrystallization processes (Figure 7, top left). As can be seen a very fine-grained microstructure could be developed through the reduction of the distance leading to a higher rolling temperature. However, the residual heat in the weld metal after the rolling process is not sufficient to enable further grain growth to achieve the desired homogeneous microstructure. Regarding the coarse grained HAZ a change from an acicular ferritic structure (Figure 7 bottom right) into a ferritic/perlitic structure (Figure 7, bottom left/middle) can be recognized. Meaning that also areas which are not directly in contact with the rollers, experience a high enough plastic strain to initiate microstructural changes. Unfortunately, there are no recrystallization phenomena present in the coarse-grained area.

**Figure 8:**
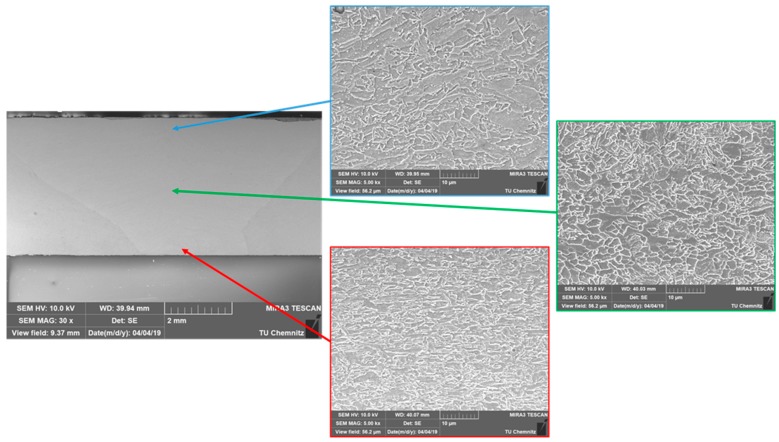
SEM images of the developing microstructures in the weld metal by a torch to roller distance of 105 mm.

Further investigations with the SEM, on a sample formed at a torch to roller distance of 105 mm, show that elongated grains are still present in the microstructure, especially in the top and the bottom part of the weld metal (Figure 8). These areas are located closest to the direct contact to the rollers, resulting in a high heat dissipation. Thus, although there is a high plastic strain, the residual heat is not high enough for a complete recrystallization.

This corresponds with the results of the numerical simulation shown in Section 4.4, in which a total fraction of recrystallization of approx. 75 % was calculated.

### 3.4. Mechanical Properties

**Figure 9:**
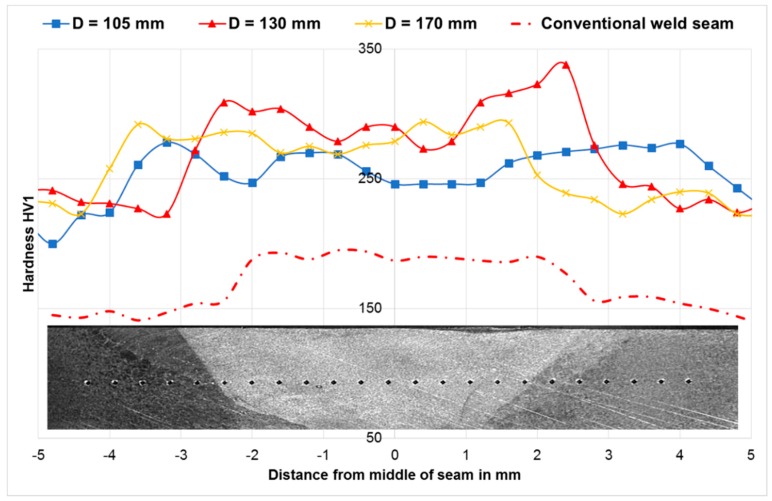
Hardness measurements of the weld metal and HAZ for different torch to roller distances.

In order to quantify the influence of the distance between torch and roll center on the mechanical properties, hardness measurements were carried out in the middle of the weld seam and the HAZ. For the Vickers hardness measurement (HV1), according to EN ISO 6507, an Emcotest Durascan G5 was used. Figure 9 shows the resulting hardness profiles of the weld metal and HAZ of the different distances compared to the hardness profile of a standard weld as a reference. In general, it can be stated that the hardness increases due to the WeldForming process both in the weld metal and in the coarse grained HAZ—independent of the distance between the welding torch and roll center. Furthermore, it can be seen from Figure 9 that the hardness at a distance of 105 mm decreases compared to the further distances. The decreased hardness can be explained by a higher fraction of recrystallized areas. This thesis has already been proven with the light microscopic and SEM investigations in Section 3.3. Nevertheless, it should be noted that the difference in hardness between the coarse grain zone and the weld seam could not be significantly reduced by welding compared to the standard welding process.

## 4. Material Modelling and Numerical Simulation

The aim of WeldForming is to prevent the typical zone formation in the welding area and achieve a homogeneous microstructure. To analyze and predict the microstructural transformations numerically, it is vital to determine flow curves and the recrystallization behavior for different initial microstructures. The initial microstructure of the samples used thereby has to be identical to the microstructures that are present in the welding area especially regarding the orientation. The reproduction of the cast-like structure of the weld metal in the correct orientation is particularly difficult. Therefore, the procedure of this sample preparation is explained in more detail in the following Section 4.1 and Section 4.2. Based on the material model the numerical model for the WeldForming process is introduced in Section 4.3 and the results are discussed in Section 4.4.

### 4.1. Sample Preparation

**Figure 10:**
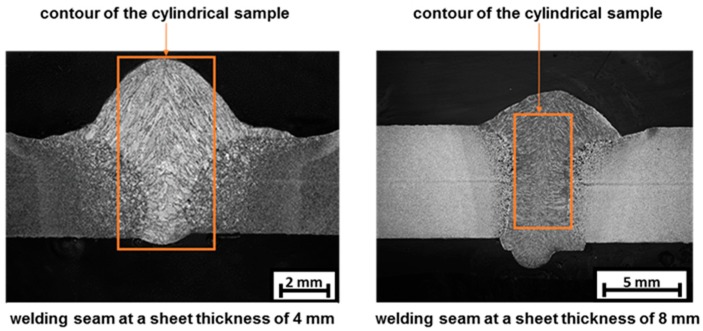
Weld seam with a sheet thickness of 4 mm and 8 mm and contour of the cylindrical specimen.

For the material characterization using the forming and quenching dilatometer “DIL 805 A/D” cylindrical specimens with minimum dimension of Ø 4x8 mm are needed. Unfortunately, it is not possible to achieve these geometric dimensions by a single layer welding procedure, as illustrated in Figure 10. In principle, it is possible to extract a sample from the weld seam using a sheet thickness of 8 mm. But in this case, there still is a mixture of base material and filler metal in the lateral area. Since the face reinforcement (pure filler material) of the seam is levelled during the WeldForming process, it is necessary to characterize the material behavior of the pure filler material. Therefore, the microstructure of the cylindrical specimens must be identical to the microstructure present in the weld seam. The general procedures for the metallographic preparation of metallic materials were used in combination with chemical etching, using Nital.

Since it is not possible to extract samples directly out of the weld seam, an alternative strategy was developed which leads to samples with the following properties:

- Samples with a height of 8 mm and a diameter of 4 mm
- Microstructural properties of the weld structure (dendritic structure)
- Cylindrical sample consists of pure filler material (no areas with material mixing)

#### New Approach

**Figure 11:**
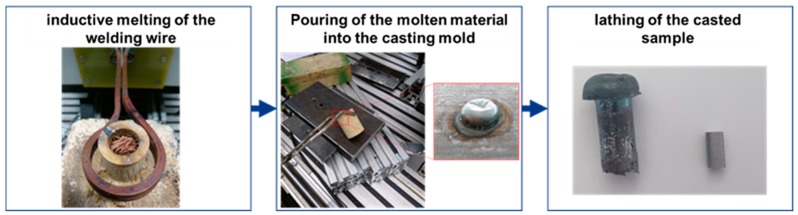
Procedure of the casting process for producing cylindrical specimens.

To achieve samples with the above-mentioned characteristics a casting method with a specifically formed mold was developed. The raw material (welding wire G4Si1) is melted by induction heating. The molten material is then poured into a metallic casting mold. Once solidified the cylindrical specimen for the upsetting test is then produced out of the casted metal by a lathing process. The procedure of the casting process is shown in Figure 11.

The characteristic dendritic microstructure of the weld metal results out of the rapid cooling of the molten material. To achieve a similar microstructure in the casting process the cooling conditions must be comparable to the welding process. In order to guarantee this, the cooling behavior of the weld seam was analyzed in a first step. For this purpose, a thermocouple type B was placed in the solidifying weld metal during the welding process and the temperature-time curve was measured. Equal measurements were then performed during the casting process.

**Figure 12:**
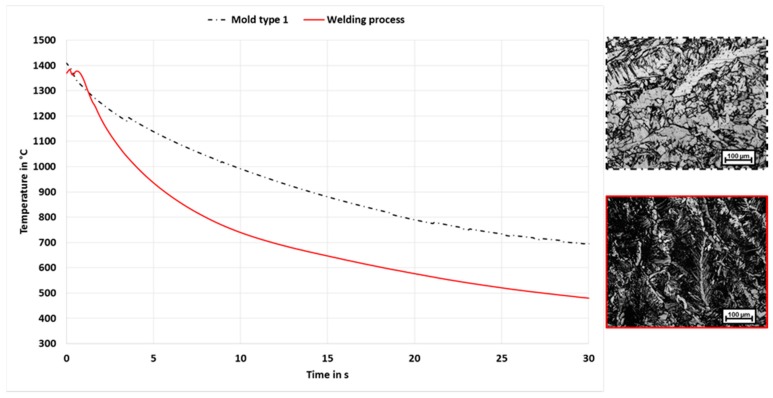
Comparison of cooling behavior and microstructure of the welding and casting process with casting mold 1.

As first approach the molten filler metal was poured into a mold with slotted holes, to maximize the sample yield. The slotted holes of the casting mold type 1 were 35 mm long, 8 mm wide and 13 mm deep. The edges were rounded with a radius of 2 mm. A comparison of the cooling behavior shows clear differences between the welding process and the casting process with the casting mold type 1. The cooling behavior and the resulting microstructure of the welding and casting process with casting mold 1 is shown in Figure 12. The t8/5-time of the welding processes is 19.4 s whereas for the casting process a t8/5-time of 39.7 s could be measured. The conclusion is that the ratio between sample volume and the surface of the contact areas is too large so that the heat of the molten material cannot be dissipated quickly enough.

**Figure 13:**
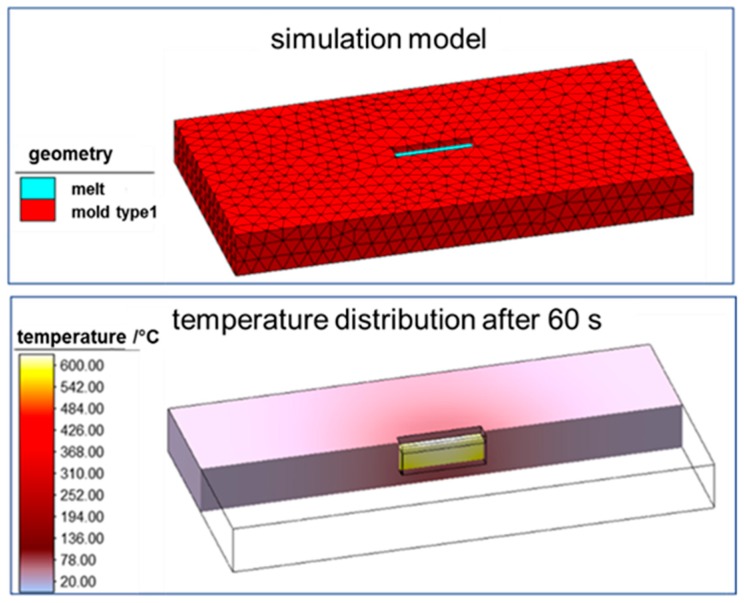
Simulation setup and simulation of the temperature distribution after 60 s.

To determine an adequate casting mold, the cooling behavior was analyzed with a numerical simulation. Within the simulation, the geometry of the casting mold could be varied at will until the cooling of the melt in the mold sufficiently matches the cooling behavior of the welding process. The molten material is defined as a separate contact body with an initial temperature of 1500 °C. At the beginning of the simulation, the contact body (molten material) is already in the casting mold, which is defined as a tool with heat conduction. Only the cooling process is calculated and not the actual pouring of the melt. This simplification is sufficient for predicting the cooling characteristics in the respective casting mold. Figure 13 shows the simulation setup and the calculated temperature distribution after a cooling time of 60 seconds. The simulation model was verified in advance on the basis of experimentally measured cooling curves of the casting mold type 1.

With the aid of the numerical simulation, a new casting mold was developed in which a cooling rate similar to the welding process could be achieved. The optimized casting mold is cylindrical with a diameter of 8 mm and a depth of 13 mm.

**Figure 14:**
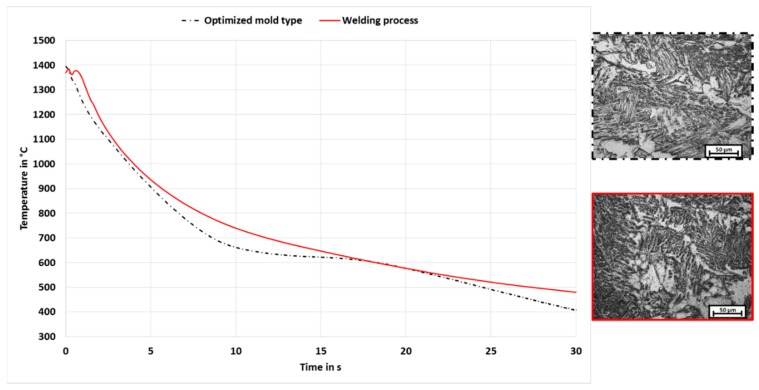
Comparison of cooling behavior and microstructure of the welding and optimized casting process.

A comparison of the cooling behavior between welding process and the casting process with the optimized mold type is shown in Figure 14. The measurements show that in a temperature range from approx. 1500 °C (melt) to 500 °C (microstructure completely developed) similar cooling conditions can be achieved. The corresponding t8/5-time is 17.85 s for the optimized casting mold and 19.4 s in the welding process. The difference with respect to the measured t8/5-times is less than 10%, so that a similar microstructure state can be assumed at room temperature. On the basis of microscopic investigations with a light microscope, the microstructure resulting out of the casting with the optimized mold type was compared to the structure of a standard weld seam. Figure 14, right shows this comparison. It can be seen that there is a high similarity of the microstructure produced by the casting technique to the microstructure in the weld seam area.

### 4.2. Physical Simulation for Material Characterization

For the material characterization upsetting tests were performed. These tests were used to determine the dynamic recrystallization behavior of the filler metal with a cast-like microstructure for the first time. The static and metadynamic behavior of the materials used, were determined by relaxation tests.

**Figure 15:**
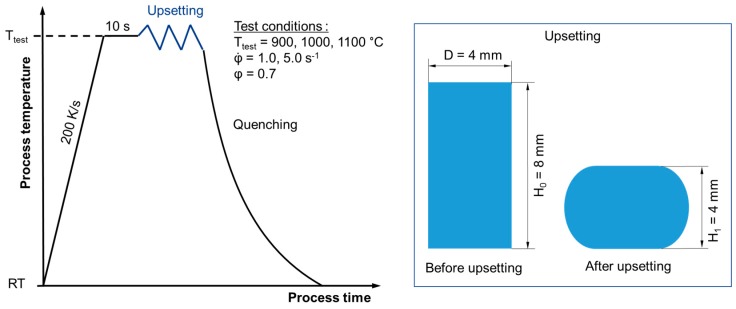
Test procedure (left) and sample geometry (right) for upsetting tests.

The cylindrical samples are inductively heated to test temperature and kept at this temperature for 10 s. After this, the specimens cool down through convective heat transfer to the desired forming temperatures. A full factorial experimental design was used in which four forming temperatures were tested (700; 900; 1000 and 1100 °C) and two constant strain rates (1 and 5 s^−1^) up to a plastic strain of 0.7. To ensure statistical verification, three tests were carried out per test condition. The test procedure for the upsetting test and the sample geometry is shown in Figure 15.

**Figure 16:**
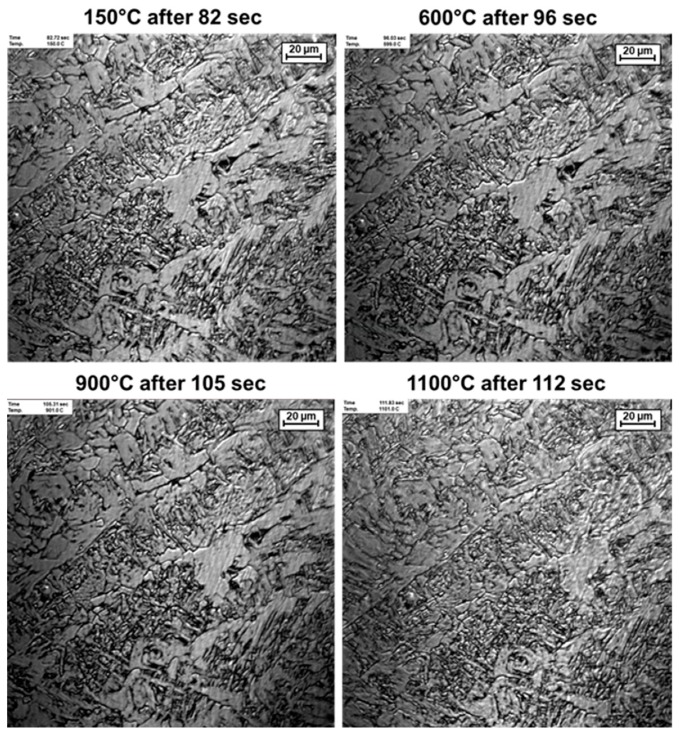
In-Situ microstructural images of a heating cycle.

The heating rate of 200 K/s is sufficient to avoid microstructural transformations as far as possible. Studies on the Yonekura In-Situ High temperature observation Confocal Scanning Laser Microscope show that even at a heating rate of 32 K/s no far-reaching transformations occur (cf. Figure 16). Only at a temperature of 1100 °C first signs of liquefying and transformation appear.

Thus the selected temperature profile is suitable to determine the necessary material data by using upsetting tests.

**Figure 17:**
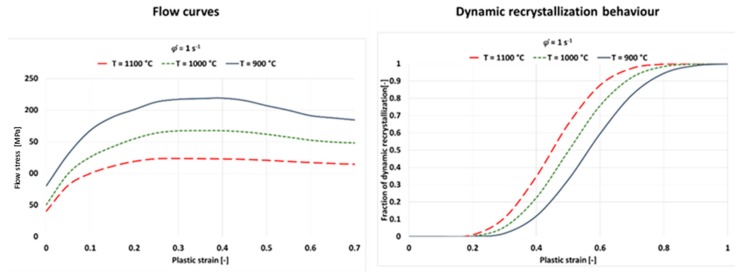
Flow curves and dynamic recrystallization for a strain rate of 1.0 s^–1^ of weld metal.

In order to consider the recrystallization kinetics within the numerical process simulation, the mathematical modeling was performed using the JMAK theory (Johnson-Mehl-Avrami-Kolmogorov theory). In a first step, temperature-dependent flow curves are determined, from which the necessary parameters for the modeling of the dynamic recrystallization can be determined. A detailed modelling procedure can be found in Ullmann (2014). For a constant strain rate of 1.0 s^−1^ and test temperatures of 1100 °C, 1000 °C and 900 °C, the flow curves and the resulting dynamic recrystallization behavior are shown in Figure 17.

### 4.3. Finte Element Model

**Figure 18:**
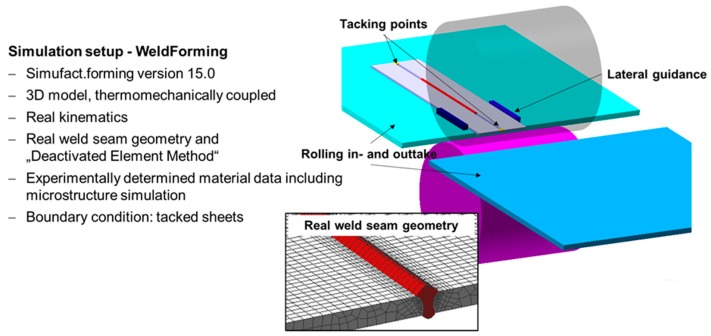
Summary of the simulation setup for WeldForming.

In order to reproduce the WeldForming process realistically, a numerical model was developed in which booth processes (welding and forming) were combined in a single simulation model, considering the occurring microstructural changes. In Härtel and Adams [16] the simulation setup as well as the validation of the welding and rolling simulation is described in detail. Figure 18 summarizes the most important geometrical and numerical features of the simulation setup for WeldForming.

### 4.4. Results and Comparing the Model with Experiments

Numerical simulation is used in the following chapter to understand which hardening and softening phenomena occur in the weld metal as well as in the coarse grained HAZ. The aim of numerical process analysis is to derive optimization potentials in order to obtain mechanical properties that are as homogeneous as possible.

**Figure 19:**
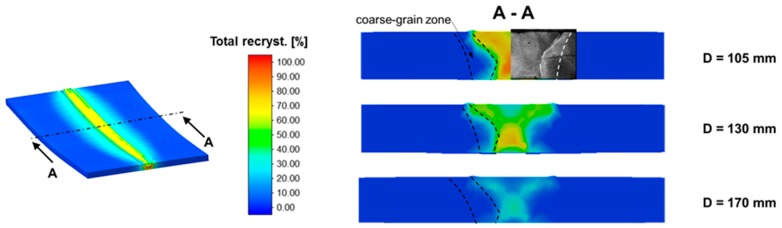
Fraction of total recrystallization depending on the distance between the torch and the roll.

At the beginning, the recrystallization behavior in the weld area was analyzed, since the primary goal of the WeldForming process is the transformation of the acicular weld structure into a globular forming structure. Figure 19 shows the total fraction of recrystallization after the WeldForming process for different distances between the torch and the roll center. At a distance of 105 mm, almost complete recrystallization is achieved in the middle of the seam area. However, an average of 75% of the microstructure recrystallizes in the seam. Increasing the distance, torch to roller, to 130 mm generally leads to a reduced recrystallization behavior of approx. 55% in the seam area. A further increase in the distance from welding torch to roller to 170 mm leads to a significant reduction in the recrystallized microstructure content to approx. 15%. Figure 19 also shows that recrystallization processes in the coarse grained HAZ are only initiated at a distance of 105 mm and 130 mm. However, this recrystallization only takes place in a small area of the coarse-grained zone and amounts to approx. 30%.

The analyzes of the fraction of total recrystallization show that the weld microstructure does not recrystallize completely under the prevailing forming conditions. The causes for this effect are subsequently analyzed by evaluating the temperature in the forming zone as well as the plastic strain distribution in the joining area after rolling.

**Figure 20:**
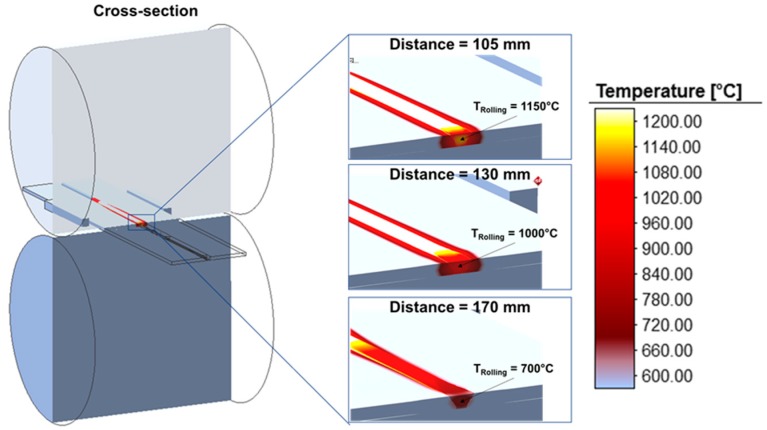
Temperature distribution in the rolling gap.

Figure 20 shows the temperature distribution of the component in the roll gap (during rolling) for different distances between the welding torch and roll center. It can be seen that with increasing distance the temperature in the roll gap decreases from 1150°C at a distance of 105 mm to 700°C at a distance of 170 mm. Due to the larger distance, the process time is longer until the weld metal is rolled, resulting in a longer time period in which the heat can dissipate to the air or the roll table. The lower forming temperature has a negative influence on the recrystallization.

**Figure 21:**
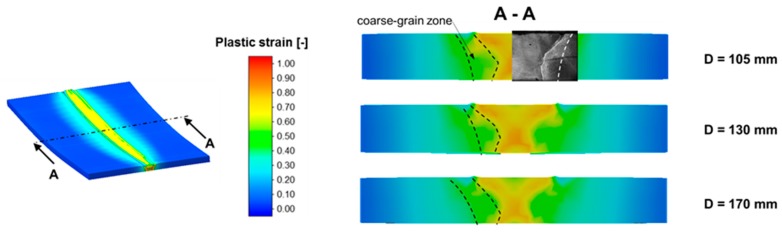
Plastic strain distribution after rolling.

Figure 21 shows the distribution of plastic strain after leveling the face reinforcement-to-sheet thickness. In principle, it can be determined that the distribution of the plastic strain is almost identical for all distances. On average, the plastic strain in the seam area is approx. 0.65. However, a slight inhomogeneity can be observed with regard to the plastic strain distribution. In the middle of the seam, the plastic strain is approx. 0.8. In combination with the increased temperature in the middle of the seam, the increased plastic strain leads to a larger fraction of recrystallized microstructure, as can be seen in Figure 19.

Unfortunately, a complete recrystallization cannot be achieved with the current experimental setup (see Figure 5). Particularly in the coarse grained HAZ, only low recrystallization effects can be observed, although a plastic strain of approx. 0.4 is achieved. This also correlates with the hardness measurements carried out in Section 3.4, which showed that the hardness in the coarse-grained zone increases as a result of the WeldForming process. At the same time, the microscopic images showed nearly no structural changes due to recrystallisation processes, so that the increase in hardness is the result of the material hardening due to the rolling process. In the following chapter a first-time process optimization is presented, with which an almost complete recrystallization in the weld metal and coarse grained HAZ can be achieved.

## 5. Modelling and Experiments with Inline-Plasma Treatment

**Figure 22:**
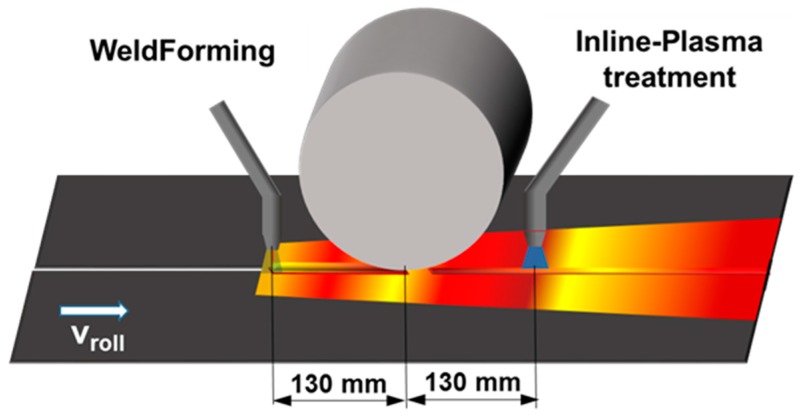
WeldForming with Inline-Plasma treatment.

Based on the previous investigations, the optimization approach for the WeldForming process is developed for a distance between the welding torch and roll center of 130 mm. The idea of the optimization is to utilize the hardening effect induced by the rolling process in order to initiate static recrystallization in areas of incomplete dynamic recrystallization by means of subsequent heating. The subsequent inline reheating unit is to be integrated into the process. The principle process chain is shown in Figure 22.

Since static recrystallization should be initiated predominantly in the weld metal and coarse-grained zone, a heat source with a thermal field of approx. 20 mm is suitable. In addition, the heat source must provide a high energy input in order to heat the material inline to a temperature of approx. 900 °C. Within the scope of this work, the possibility of a plasma torch as a post-heating unit is investigated

In a first step, the existing simulation model is extended by a volumetric heat source that simulates the heat input through the plasma torch. The used parameters of the heat source are summarized in Table 2. The distance from the roll center to the heat source of the plasma torch is 130 mm for initial investigations (see Figure 22).

**Figure 23:**
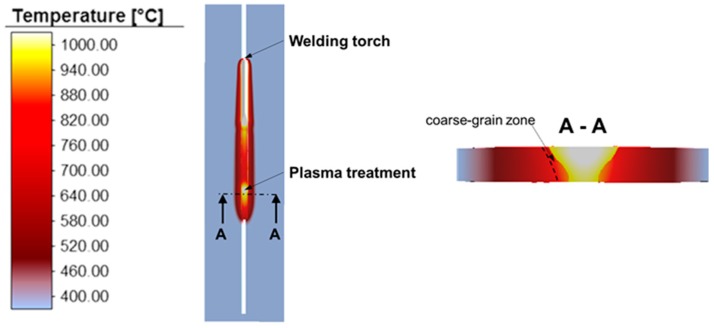
Temperature distribution for WeldForming with Inline-Plasma treatment.

As a result of the post heating with a plasma torch, the temperature distribution in the work piece changes as shown in Figure 23. In the area of the weld metal, a temperature of approx. 1000 °C is reached with the above-mentioned parameters for the heat source. In the coarse grain zone, over 800 °C is reached.

**Figure 24:**
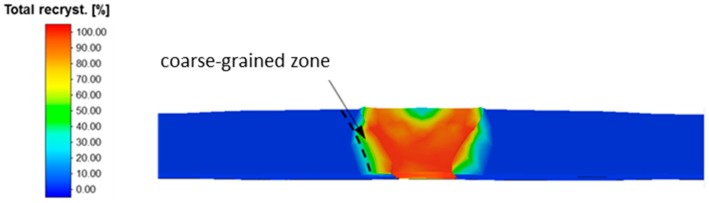
Fraction of total recrystallization after WeldForming with Inline-Plasma treatment.

The additional heating leads to an almost complete recrystallization in the weld metal, as shown in Figure 24. Further, in the area of the coarse grained HAZ approx. 60% of the microstructure recrystallized. The basic functional proof, that an inline coupling with an additional post-heating unit improves the recrystallization behavior in the weld seam as well as in the coarse grain zone could be numerically proven.

The experimental implementation of the post-heating unit has not yet been realized with the current machine design. However, the experimental functional proof was carried out by heat-treating samples produced by WeldForming with a plasma torch. In preliminary tests, a suitable distance from plasma torch to sheet metal and suitable heat-treating parameters were determined so that a maximum temperature of 1000 °C is not exceeded.

**Figure 25:**
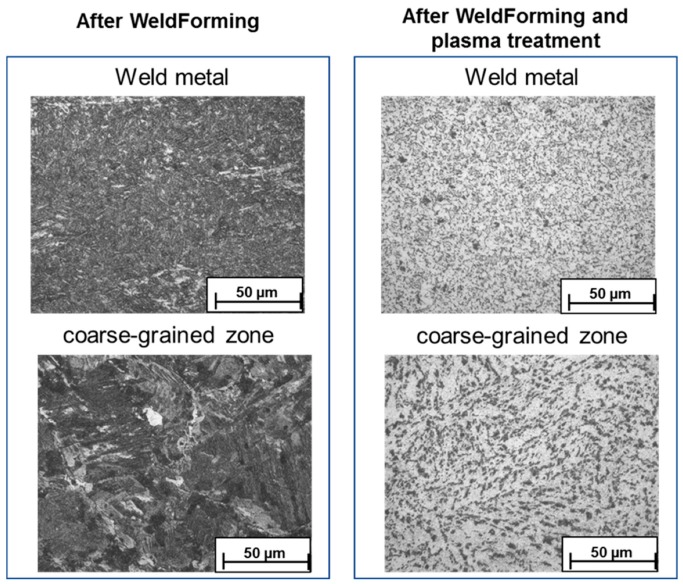
Micrographs of the weld metal and the coarse-grained zone after WeldForming and after WeldForming with plasma post-treatment.

The influence of the plasma post-treatment on the microstructure could be quantified by light microscopy. Figure 25 shows micrographs of the weld metal and the coarse-grained zone after WeldForming and after WeldForming with plasma treatment. In the area of the weld metal, the plasma post-treatment leads to a very fine-grained and homogeneous microstructure with an average grain size of approx. 5 µm. In the coarse-grained zone, a grain refinement could also be achieved. However, a slight texture is still visible in the microstructure, which suggests that no complete recrystallization could be achieved just yet.

**Figure 26:**
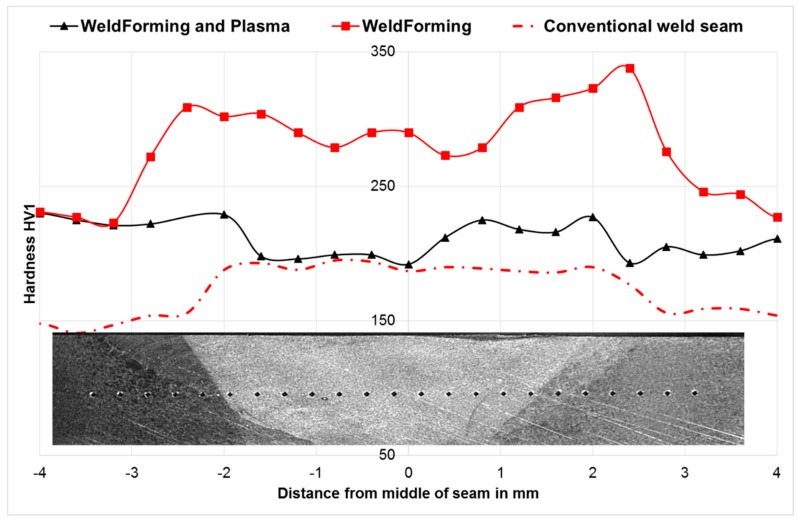
Hardness of weld seam and coarse grain zone for different welding methods.

In addition to the micrographs, the influence of plasma post-treatment on the mechanical properties was quantified by hardness measurements. Figure 26 shows the hardness profile in the weld metal and coarse-grained zone. It can be seen that the plasma post-treatment leads to a homogenization of the mechanical properties. The hardness in the weld metal is reduced to approx. 210 HV1, which corresponds to the hardness of a conventional seam. It can therefore be assumed that the hardening effect of the forming process could be reduced. In the coarse-grained area, the plasma post-treatment does not lead to any significant reduction in hardness compared to the WeldForming process. One explanation could be that recrystallization degrades a fraction of the hardening effect, resulting in a reduction of hardness. At the same time, recrystallization in the coarse-grained zone leads to grain refinement, which in turn leads to an increase in hardness. To which extent these two mechanisms influence each other will be investigated in upcoming studies.

Nevertheless, the following improvements could be achieved through the process optimization:

- complete recrystallization in the joining area and transformation of the weld structure into a fine-grained globular structure
- grain refinement in the coarse-grained zone by initiation of recrystallization
- reduction of the hardness differences between coarse-grained zone and weld metal and thus homogenization of the mechanical properties

In continuing work, the inline process will also be experimentally implemented and further optimized with the aim of complete recrystallization in the HAZ.

## 6. Conclusions

In this paper a novel method for altering the microstructure of a welded joint was introduced. Through the coupling of a welding and a rolling process, a microstructure modification could be achieved by means of dynamic, metadynamic and static recrystallization phenomena. Further, a new approach for manufacturing samples with similar microstructure, orientation and properties of the solidified weld metal was presented. To achieve the comparable microstructure, the casting mold geometry was optimized numerically and validated by experimental investigations. In initial investigations, the flow curves and recrystallization kinetics were determined from upsetting and relaxation tests in order to describe the material behavior of the filler material in the welding zone. The gathered data is used for numerical process coupling of a welding and forming process, which was done in a single simulation model for the first time. The simulation model is based on verified data of the individual processes, taking into account the microstructural transformations in the welding area. Based on this, the influence of the distance between the welding torch and the center of the rolls on the plastic strain, the forming temperature and the strain rate was analyzed to evaluate the recrystallization effects. On the basis of the numerical calculations, the WeldForming process was implemented experimentally. The possibility to transform a cast-like and brittle microstructure into a recrystallized grain structure as a result of the coupled process setup was proven. To achieve a complete recrystallization and a homogeneous microstructure in the whole joining area an optimization approach was presented. With the aid of an additional inline post heat treatment a further increase of recrystallized areas could be reached.

Further work will investigate methods to reduce the cooling rate during the forming process, to reduce residual stresses, stimulate the grain growth and achieve a homogeneous microstructure over the whole welding area.

## 7. Patents

DE102015116191A1—Verfahren zur umforminduzierten Schweißnahtbehandlung (engl.: Method for forming-induced weld seam treatment).

## Figures and Tables

**Table 1:** Nominal chemical composition of the base material and the filler material (wt%).

Material	Fe	C	Mn	Si	P	S
S235JR (base material)	Balance	<0.2	<1.4	-	<0.045	<0.045
G4Si1 (filler material)	Balance	0.08	1.5	0.9	<0.025	<0.025

**Table 2:** Parameters of the heat source for subsequent heating.

Parameter	Value
Power	1.4 kW
Efficiency	85%
Width	8 mm
Depth	12 mm
